# Stressor Complexity, Ecological Response, and Conservation Decisions: Advances in Wildlife Conservation and Habitat Management in the Anthropocene

**DOI:** 10.3390/biology15131057

**Published:** 2026-07-02

**Authors:** Yiannis G. Zevgolis, Panayiotis G. Dimitrakopoulos

**Affiliations:** Biodiversity Conservation Laboratory, Department of Environment, University of the Aegean, 81132 Mytilene, Greece

## 1. Introduction

Wildlife conservation and habitat management have entered a phase of irreducible complexity, in which the ecosystems where species must persist, populations must recover, and management must intervene are increasingly structured by interacting constellations of anthropogenic, biotic, and abiotic change [1,2]. Land-use transformation, habitat fragmentation, altered disturbance regimes, agricultural intensification, biological invasions, infrastructure expansion, overexploitation, and climate change now operate simultaneously across biological, temporal and spatial scales [3,4], generating ecological outcomes that are nonlinear, taxon-specific, historically contingent, and spatially heterogeneous in ways that linear or single-driver frameworks are poorly equipped to anticipate [5,6,7]. The defining conservation challenge of the Anthropocene therefore lies in the capacity to organise ecological knowledge in ways that can navigate complexity: characterising how stressors interact, detecting the biological responses through which species and communities register those interactions, translating those responses into spatial or demographic diagnosis, and sustaining the adaptive feedback between management action and ecological outcome on which effective conservation ultimately depends.

The 16 contributions assembled in this Special Issue collectively address this challenge, expressed through the full analytical passage from stressor characterisation to implemented, monitored, and revisable management action. The collection extends across terrestrial, insular, freshwater, agroecological, protected-area, and human-modified systems, addressing conservation problems that range from the continental-scale connectivity crisis facing the clouded leopard (*Neofelis nebulosa*) [8], to the intensely localised convergence of seasonal water scarcity, rising summer temperatures, and intensified predation threatening *Pelophylax cerigensis*—Europe’s most threatened frog—within the restricted freshwater systems of an Aegean island [9]. These seemingly distant cases are joined by a shared evidentiary demand: to characterise interacting stressors with ecological precision, to measure the biological responses through which species and communities register those pressures, and to convert that evidence into decisions that are spatially explicit, adaptive, and accountable through future monitoring.

**Figure 1:**
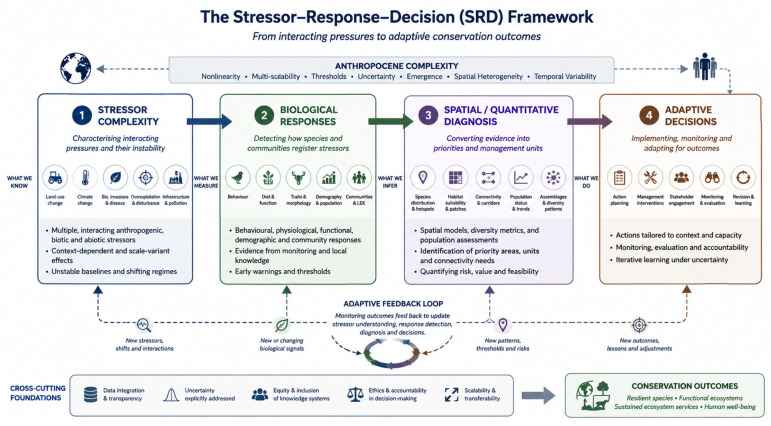
The Stressor–Response–Decision (SRD) framework for conservation science under Anthropocene complexity. The framework conceptualises conservation evidence as a connected circuit linking interacting anthropogenic, biotic, and abiotic pressures to biological responses, spatial or quantitative diagnosis, and adaptive conservation decisions. Stressor characterisation identifies the pressures and interactions structuring ecological systems; response measurement detects how species, populations, communities, and knowledge systems register those pressures; diagnosis translates evidence into hotspots, habitat patches, corridors, population status, assemblage patterns, and management units; and adaptive decisions implement, monitor, evaluate, and revise conservation action. The feedback loop emphasises that monitoring outcomes should update stressor understanding, response detection, diagnostic priorities, and management decisions, maintaining the integrity of the full evidence-to-decision chain.

Together, the contributions advance the central argument of this editorial: wildlife conservation becomes most consequential when stressor complexity, ecological response, and conservation decision are organised within a single chain of evidence, capable of guiding action while remaining open to monitoring, challenges, and revision. This chain provides the basis for the Stressor–Response–Decision (SRD) framework (Figure 1) developed below, through which the 16 contributions are read as connected points along the passage from interacting pressures to biological response, from response to spatial or demographic diagnosis, and from diagnosis to adaptive conservation action. Following this introductory section, this editorial develops this passage through four analytical arcs— the instability of conservation assumptions under interacting pressures; the biological responses through which those pressures become measurable; the spatial and quantitative diagnoses that convert ecological evidence into prioritisation; and the translation of diagnosis into adaptive, accountable action—before returning to the SRD framework as a synthetic argument for conservation science under Anthropocene complexity.

## 2. Stressor Complexity and the Fragility of Conservation Assumptions

The first thematic arc of the SRD framework begins with the recognition that Anthropocene pressures do not merely intensify conservation problems; they also destabilise the conceptual categories through which those problems are identified, compared, and managed. Disturbance, recovery, mitigation, management, and prediction remain central to conservation thought, yet each carries an assumed ecological direction that interacting pressures can unsettle: disturbance should explain damage, recovery should mark improvement, mitigation should reduce risk, management should restore control, and prediction should make the future more legible. The contributions considered here show that these expectations acquire conservation meaning only when they are tested against biological response, landscape configuration, temporal contingency, and the historical conditions under which ecological processes unfold. Under Anthropocene complexity, a pressure becomes conservation-relevant through the measurable consequences it produces within a particular system, and the analytical task is therefore to identify when a process becomes a threat, a resource, a misleading signal, or a management opportunity.

This problem is especially visible in landscapes where disturbance and land use operate as structuring forces rather than discrete external events. In Mediterranean rangelands, livestock grazing generated divergent vertebrate responses across abundance, species richness, vegetation structure, and resource availability, showing that grazing intensity acquires ecological meaning only through the multiple response axes along which communities reorganise [10]; a prescription calibrated to richness alone may therefore misread abundance, vegetation structure, or resource supply. Fire poses a parallel challenge in post-disturbance wetlands, where burned and unburned patches function less as fixed habitat categories than as a spatially and temporally dynamic resource mosaic in which predator and scavenger activity is reorganised by season, prey exposure, carrion availability, and trophic opportunity [11]. In both cases, disturbance is transformed from a management label into an ecological question: its conservation meaning emerges from the biological response it elicits, the timing of that response, and the spatial configuration through which disturbance becomes constraint, release, or reorganisation.

Recovery introduces an even sharper test, because it is among the most intuitively powerful assumptions in applied conservation and therefore among the most consequential when it is treated as self-evident. The study of South China sika deer (*Cervus nippon kopschi*) over a decade of active forest restoration demonstrates that vegetation regeneration may advance structural and spectral indicators of recovery while simultaneously failing to improve functional habitat use by the target herbivore [12]. This distinction is central to conservation in recovering landscapes: greenness, canopy development, and vegetation density may describe the trajectory of the habitat while obscuring the ecological experience of the organism moving through it. A restored landscape can therefore be treated as biologically recovered only when structural change is confirmed through species use and functional habitat suitability. Otherwise, restoration remains a structural success whose conservation value is assumed rather than empirically demonstrated.

The decoupling between apparent and functional success is equally visible in conservation intervention and predictive modelling. Sustainable management of the olive fly (*Bactrocera oleae*) cannot be reduced to pest suppression alone, because host phenology, climatic sensitivity, natural enemies, pesticide resistance, and landscape structure all shape whether control is durable or ecologically costly [13]. Insecticide applications may reduce pest pressure while disrupting beneficial organisms and weakening the biological regulation they replace; climate change further widens this uncertainty by altering the timing, intensity, and predictability of olive fly pressure. The review’s call for ecologically informed management is therefore also a conservation argument: in the Anthropocene, where agricultural landscapes occupy vast areas and interact continuously with surrounding biodiversity, pest management policy is biodiversity policy.

Predictive modelling faces a parallel demand for accountability. The retrospective evaluation of Egyptian vulture (*Neophron percnopterus*) projections under wind-farm mortality shows that the conservation authority of models depends on whether their assumptions are tested against the trajectories they claim to anticipate [14]. Short demographic baselines, weakly calibrated exposure estimates, and untested projections allow forecasts to influence regulation without being exposed to the consequences of error. Prediction, like disturbance, recovery, and management, becomes conservation-relevant only when it remains accountable to biological response. Identifying when and how that accountability is met, however, requires a different order of evidence: the biological responses through which species and communities make stressor effects visible, measurable, and interpretable at the resolution that management decisions demand.

## 3. Biological Responses and the Precision of Conservation Evidence

The second thematic arc of the SRD framework rests on the recognition that if interacting pressures destabilise the assumed meaning of disturbance, recovery, management, and prediction, then biological response becomes the point at which conservation interpretation regains precision. Species and communities register Anthropocene pressures through behavioural thresholds, dietary adjustments, trait variation, demographic structure, and the uneven ways vulnerability is expressed across biological scales. The response component of the SRD framework therefore shifts conservation evidence away from the question of whether a species is simply still present, and toward the more challenging question of how it is persisting, under what constraints, and with what hidden biological costs.

Behaviour is often the first place where pressure becomes measurable, because animals alter space use, escape decisions, and tolerance before changes in population size can be detected. Husby [15], through the reassessment of flight initiation distance in birds, shows that disturbance cannot be reduced to a generic buffer distance or average escape response; it must be interpreted through species identity, flock size, disturbance type, site context, and the probability structure of escape behaviour. The management value of behavioural metrics therefore lies in their capacity to preserve the variability through which animals actually experience risk, allowing disturbance thresholds to be designed from biological sensitivity instead of administrative convenience.

Dietary evidence carries the same logic into the functional dimension of habitat quality. In anthropogenic landscapes, the persistence of European ground squirrels cannot be inferred from occurrence alone, because feeding habits reveal the resource pathways through which animals negotiate modified habitats [16]. Diet becomes a diagnostic interface between habitat structure and biological requirement, showing whether a species is exploiting available resources, compensating for scarcity, narrowing its trophic options, or being pushed toward potentially suboptimal foods. In the populations studied, the use of plants with potentially adverse secondary compounds turns feeding ecology into evidence of functional constraint, converting the broad category of human-modified habitat into the more exact question of whether that habitat remains biologically sufficient.

Trait and demographic evidence deepen this response-based view by exposing forms of vulnerability that distributional data cannot resolve. The long-term morphometric assessment of sexual body size dimorphism in small mammals by Balčiauskas and Balčiauskienė [17] provides a baseline through which population structure, ecological variation, and future change can be interpreted with greater biological resolution. Tella et al. [18] extend this logic into demographic hiddenness, showing that conservation assessment of the Red-spectacled Amazon depends on distinguishing breeding and non-breeding fractions instead of treating visible abundance as demographic security. A population may remain numerically present while being structurally constrained; the conservation signal lies not only in how many individuals exist, but in how those individuals are organised, recruited, and able to sustain future persistence.

Local ecological knowledge expands this evidentiary architecture by capturing population visibility where conventional monitoring is logistically difficult, temporally sparse, or financially constrained. Wenborn et al. [19] show that structured knowledge from community game guards can support meaningful inference on elephant population size in remote landscapes, functioning as an organised observational system grounded in repeated encounter, spatial familiarity, and local continuity. Where survey capacity is absent, such systems extend the geographic reach of the evidentiary architecture that biological response monitoring requires.

## 4. Spatial Diagnosis and Conservation Prioritisation

The third thematic arc of the SRD framework begins at the point where biological response evidence must be converted into conservation choice. A behavioural threshold, a dietary constraint, a demographic imbalance, a locally observed population estimate, or a community-level shift becomes operational only when conservation science can locate it, compare it, and determine where action will matter most. Diagnosis therefore does more than map species or quantify their status; it identifies where vulnerability is concentrated, where ecological value and management conflict overlap, where future persistence depends on connectivity, and where limited conservation resources can be directed with the greatest justification.

This conversion of evidence into decision space is especially clear in the European rabbit study on Lemnos Island, where nearly 60% of high-density areas of *Oryctolagus cuniculus* coincide with formally designated conservation zones [20]. This overlap should be read as a diagnostic signal of how insular agroecosystems concentrate ecological value, population density, land-use context, and management conflict within the same landscape units. By combining 1534 occurrence records with environmental drivers, hotspot analysis, and spatial modelling, the study transforms distributional information into a map of management consequence, showing that the conservation relevance of a species is produced not by presence alone, but by where that presence intersects with institutional obligations, ecological function, and competing land-use demands.

Where the *Oryctolagus cuniculus* study diagnoses conservation conflict through concentration, studies of threatened and low-density species diagnose vulnerability through rarity, fragmentation, and spatial continuity. Khattak et al. [21] combine field evidence of Indian pangolin (*Manis crassicaudata*) presence with habitat suitability modelling to identify habitat patches of heightened conservation concern within a fragmented landscape, showing how sparse signs, local habitat structure, and predictive mapping integrate into a practical prioritisation framework. Abedin et al. [8] extend this diagnostic logic to a continental scale for the clouded leopard (*Neofelis nebulosa*), where habitat suitability forecasting, climate projections, and corridor identification reveal that conservation priority extends beyond occupied habitat to the spatial continuity required for persistence across fragmented Asian landscapes. One diagnosis works through localised patch concentration, the other through transboundary connectivity; both demonstrate that vulnerability produces conservation obligation only when its spatial structure is made explicit.

Freshwater systems complicate this diagnostic movement because what requires diagnosis is often the reorganisation of whole assemblages under cumulative pressure rather than the status of a single population. In the Pearl River Basin, He et al. [22] use zeta diversity and cumulative-effect analysis to show that human pressure reshapes fish communities through compositional turnover, uniqueness, and homogenisation that conventional richness metrics may not detect, shifting diagnosis to a comparative, community-level operation that identifies where ecological distinctiveness persists and where management should protect the spatial structure of biodiversity itself.

A parallel challenge emerges when the limiting factor is data scarcity rather than spatial complexity. The stock assessment of noncommercial fishes in Aras Dam Reservoir shows that sparse fisheries data can still be converted into management-relevant status, revealing depleted or unsustainable conditions in species that would otherwise remain outside conservation attention because they lack commercial prominence [23]. Diagnostic modelling here performs a corrective function: it prevents low visibility from being mistaken for low vulnerability.

Spatial diagnosis, in this sense, is the process by which conservation science decides where evidence becomes obligation. The SRD framework turns toward its decision component at this point: once vulnerability has been diagnosed, conservation must determine how that diagnosis becomes adaptive action, and how action remains accountable to the biological outcomes it was designed to secure.

## 5. From Diagnosis to Adaptive, Accountable Conservation Action

The fourth and final thematic arc of the SRD framework begins when diagnosed vulnerability becomes a demand for intervention. Spatial diagnosis identifies where conservation concern is concentrated, but its value is ultimately tested where evidence enters practice: when a hotspot becomes a management unit, a corridor becomes a planning priority, a depleted stock becomes a regulatory concern, or a threatened population becomes the focus of a coordinated recovery strategy. Decision-making is therefore the point at which conservation science becomes most exposed, because evidence is no longer judged only by analytical precision, but by its capacity to support actions that alter ecological trajectories.

The action plan for the Karpathos water frog, *Pelophylax cerigensis*, provides the clearest expression of this transition [9]. The species is confined to the restricted freshwater network of the island of Karpathos, where spatial limitation, seasonal water scarcity, rising summer temperatures, and predation pressure render generic prescriptions ecologically inadequate. Diagnosis must therefore become operational, with breeding sites protected, hydrological function secured, immediate pressures reduced, and monitoring sustained long enough to detect whether intervention is producing biological improvement. The action plan’s value lies in this conversion of ecological understanding into a revisable conservation sequence organised around the persistence of a species whose margin for error is exceptionally small.

Adaptive management, in this sense, is the conservation form of accountability: intervention designed with enough ecological specificity to be implemented, enough monitoring structure to be evaluated, and enough institutional flexibility to be revised. The SRD framework reaches its practical conclusion here: conservation decisions become scientifically credible when they preserve the connection between the pressures that justify action, the responses that reveal ecological effect, and the monitored outcomes that determine whether management has altered the biological trajectories it was designed to change.

## 6. The Stressor–Response–Decision Framework: Synthesis and Future Directions

The four thematic arcs traced in the preceding sections are not sequential stages in a linear workflow, but interdependent analytical movements whose conservation value derives from their connection (Figure 1). Stressor characterisation without response measurement risks producing threat catalogues rather than management evidence; response measurement without spatial diagnosis produces biological knowledge without decision space; spatial diagnosis without adaptive action produces prioritisation without consequence; and adaptive action without monitoring produces intervention without accountability. The SRD framework is therefore best understood as a conservation circuit whose utility depends on maintaining the evidentiary links between interacting pressures, biological responses, spatial or demographic diagnosis, and revisable management action.

This circuit also clarifies what remains unfinished. Models require retrospective validation; restoration must be tested against organismal use rather than structural recovery alone; monitoring must move beyond abundance to behavioural, functional, demographic, and community-level responses; spatial prioritisation must be connected to institutions capable of acting on it; and action plans must remain open to ecological feedback. The contributions assembled in this Special Issue show progress across each of these fronts, but they also reveal that conservation effectiveness depends less on excellence within any single evidentiary domain than on integration across the whole chain.

The SRD framework makes no claim to resolve the complexity of Anthropocene conservation. Its value is more practical and more precise: it makes the structure of that complexity legible. By linking stressors to responses, responses to diagnosis, and diagnosis to adaptive action, it offers a way to organise conservation knowledge without dismissing the ecological uncertainty that management must confront.
